# Thromboembolism after treatment with 4-factor prothrombin complex concentrate or plasma for warfarin-related bleeding

**DOI:** 10.1007/s11239-022-02695-5

**Published:** 2022-08-19

**Authors:** Alan S. Go, Thomas K. Leong, Sue Hee Sung, Rong Wei, Teresa N. Harrison, Nigel Gupta, Nicole Baker, Brahm Goldstein, Quazi Ataher, Matthew D. Solomon, Kristi Reynolds, for the REVERSAL Study

**Affiliations:** 1 Division of Research, Kaiser Permanente Northern California, 2000 Broadway, Oakland, CA 94612 USA; 2 Department of Health Systems Science, Kaiser Permanente Bernard J. Tyson School of Medicine, Pasadena, CA USA; 3 Departments of Epidemiology, Biostatistics and Medicine, University of California, San Francisco, CA USA; 4 Department of Medicine, Stanford University, Palo Alto, CA USA; 5 Department of Research and Evaluation, Kaiser Permanente Southern California, Pasadena, CA USA; 6 Department of Cardiac Electrophysiology, Southern CA Permanente Medical Group, Los Angeles, CA USA; 7 Clinical Epidemiology, CSL Behring, King of Prussia, PA USA; 8 Department of Cardiology, Kaiser Permanente Oakland Medical Center, Oakland, CA USA

**Keywords:** Prothrombin complex concentrate, Plasma, Bleeding, Thromboembolism, Death

## Abstract

Limited data exist in large, representative populations about whether the risk of thromboembolic events varies after receiving four-factor human prothrombin complex concentrate (4F-PCC) versus treatment with human plasma for urgent reversal of oral vitamin K antagonist therapy. We conducted a multicenter observational study to compare the 45-day risk of thromboembolic events in adults with warfarin-associated major bleeding after treatment with 4F-PCC (Kcentra®) or plasma. Hospitalized patients in two large integrated healthcare delivery systems who received 4F-PCC or plasma for reversal of warfarin due to major bleeding from January 1, 2008 to March 31, 2020 were identified and were matched 1:1 on potential confounders and a high-dimensional propensity score. Arterial and venous thromboembolic events were identified up to 45 days after receiving 4F-PCC or plasma from electronic health records and adjudicated by physician review. Among 1119 patients receiving 4F-PCC and a matched historical cohort of 1119 patients receiving plasma without a recent history of thromboembolism, mean (SD) age was 76.7 (10.5) years, 45.6% were women, and 9.4% Black, 14.6% Asian/Pacific Islander, and 15.7% Hispanic. The 45-day risk of thromboembolic events was 3.4% in those receiving 4F-PCC and 4.1% in those receiving plasma (P = 0.26; adjusted hazard ratio 0.76; 95% confidence interval 0.49–1.16). The adjusted risk of all-cause death at 45 days post-treatment was lower in those receiving 4F-PCC compared with plasma. Among a large, ethnically diverse cohort of adults treated for reversal of warfarin-associated bleeding, receipt of 4F-PCC was not associated with an excess risk of thromboembolic events at 45 days compared with plasma therapy.

**Supplementary Information:**

The online version contains supplementary material available at 10.1007/s11239-022-02695-5.

## Highlights

- The 45-day risk of thromboembolism after receiving reversal therapy for major warfarin-related bleeding was 4.0% (95% CI 3.3–4.9%).
- There was no significant adjusted difference in the 45-day risk of thromboembolism after receiving 4F-PCC vs. plasma.
- The adjusted risk of dying within 45 days post-treatment was lower in those receiving 4F-PCC compared with a historical plasma-treated cohort.
- Reversal of warfarin-related bleeding using 4F-PCC was associated with a similar short-term risk of arterial and venous thromboembolic complications compared with use of plasma in a real-world population.

## Introduction

Four-factor human prothrombin complex concentrate (4F-PCC) was approved by the U.S. Food and Drug Administration (FDA) in 2013 for urgent reversal of acquired coagulation factor deficiency induced by oral vitamin K antagonist (VKA) therapy in adults hospitalized with major bleeding. However, many patients being chronically treated with oral VKA therapy have conditions that predispose them to experience arterial or venous thromboembolic events (TEE). Therefore, acutely reversing VKA therapy puts such patients at a subsequent increased short-term risk of experiencing a TEE, and this risk may vary depending on the reversal strategy used.

In a randomized trial of 212 patients comparing 4F-PCC with plasma therapy as acute VKA reversal strategies, post-treatment TEE occurred at a low frequency overall [1]. 8 (7.8%) TEE occurred in 103 participants receiving 4F-PCC compared with 7 (6.4%) TEE in 109 participants receiving plasma within 45 days after treatment. In another randomized trial of 181 patients requiring urgent VKA reversal before a surgical or invasive procedure, 6 (7%) of those receiving 4F-PCC and 7 (8%) of those receiving plasma experienced a TEE [2]. However, low event rates and modest sample sizes in existing trials preclude a definitive assessment about TEE risk associated with 4F-PCC compared with plasma. In addition, previous studies examining the possible risk of TEE associated with use of PCC therapies outside of trials reported a relatively low incidence of TEE overall, along with no significantly increased risk of TEE compared with other therapies [3–9]. Yet, existing studies were limited by modest sample sizes, restricted participant diversity, variable PCC and comparator groups, differential follow-up, and limited adjustment for potential confounders and selection bias [3, 7].

We addressed this knowledge gap through a multicenter observational study of the contemporary, short-term risks of TEE in a large, matched, real-world cohort of adults receiving 4F-PCC or plasma for VKA-associated major bleeding.

## Methods

### Source population

The source population was based in Kaiser Permanente Northern California (KPNC) and Kaiser Permanente Southern California (KPSC), two integrated healthcare delivery systems providing inpatient, emergency and outpatient care for > 9 million members through 36 medical centers and > 490 offices. Both healthcare systems’ membership are highly representative of the California statewide population in terms of age, gender, race and socioeconomic status [10, 11]. Nearly all aspects of care are captured through an electronic health record (EHR) system integrated across all settings, with key variables extracted and standardized for research in the Kaiser Permanente Virtual Data Warehouse (VDW) [12, 13].

This study was approved by the KPNC and KPSC institutional review boards. We obtained a waiver of informed consent as the risk to patients was considered minimal given the nature of this retrospective data-only study.

### Eligibility

We initially identified all adult patients who received plasma for VKA reversal due to hospitalized bleeding between January 1, 2008 and March 31, 2020, or who received 4F-PCC for VKA reversal due to hospitalized bleeding between January 1, 2014 and March 31, 2020, using medication administration records. A bleeding event was considered major if the patient received ≥ 2 units of transfused red blood cells; receipt of a procedural intervention to treat bleeding; bleeding occurring in a critical anatomic location (i.e.., intracranial, ocular, retroperitoneal, hemopericardium or hemothorax) or that resulted in death. We excluded patients aged < 18 years, had unknown sex, had < 12 months of continuous health plan membership, had a history of TEE within 90 days before receipt of acute VKA reversal therapy, received VKA reversal therapy due to trauma or surgery, or received both 4F-PCC and plasma during the same hospitalization.

### Exposure

Our primary exposure was receipt of 4F-PCC or plasma for acute VKA reversal therapy. Patients who received plasma were further categorized as either historical patients if their index date occurred before FDA approval of Kcentra® (i.e., 2008–2013), or contemporary if their index date occurred after FDA approval of Kcentra® (2014–2020). The date of receipt of 4F-PCC or plasma was assigned as the index date.

### Follow-up and outcomes

Follow-up occurred from index date for up to 45 days after receipt of VKA reversal therapy or the first occurrence of death or health plan disenrollment, if earlier.

Our primary outcome was occurrence of acute TEE, which included venous thromboembolism (i.e., deep vein thrombosis, pulmonary embolism, other venous thromboembolism) and arterial thromboembolism (i.e., acute myocardial infarction, unstable angina, ischemic stroke or transient ischemic attack, and other acute non-coronary arterial thromboembolism). We initially searched EHR data for hospitalizations (including the index hospitalization) and emergency department visits for *International Classification of Diseases*, *Ninth Revision* (ICD-9) or *Tenth Revision* (ICD-10) discharge codes corresponding to each outcome (codes available on request). Board-certified physicians next adjudicated all potential TEE outcomes through manual review of EHR data using standardized diagnostic criteria (Supplemental Table 1). Physician reviewers were blinded to both the VKA reversal therapy received and date of the potential event by being only provided a redacted PDF to review. As a secondary outcome, we also identified all-cause death using a combination of EHR data (which includes member proxy reporting), Social Security vital status information and state death certificate data [14].

### Covariates

We obtained EHR data on demographics, comorbidities, laboratory results, medication use, and vital signs using *ICD-9/10* and *Current Procedural Terminology* codes as well as corresponding EHR-based data elements using validated algorithms [15–17].

### Matching

To enhance comparability between 4F-PCC and plasma patients, we created a cohort of patients receiving 4F-PCC matched 1:1 to historical patients receiving plasma on age (< 65, 65–79, and ≥ 80 years), sex (female vs. male), type of bleed (intracranial vs. extracranial), and a high-dimensional propensity score (hd-PS) [18] for receipt of 4F-PCC. To generate the hd-PS, we performed multivariable logistic regression for predicting receipt of 4F-PCC (vs. plasma) among 4F-PCC and contemporary plasma patients using patient demographics and pre-admission diagnoses, procedures and prescription medications, with variables selected by an algorithm that identified and prioritized candidate variables based on the empirical association between the candidate variable and the event. The final hd-PS model included 300 algorithmically selected variables and showed excellent model discrimination (c-statistic 0.83). We then applied the resulting model to the historical plasma-treated patients. After the hd-PS was generated for each patient, we implemented individual-level matching based on the nearest neighbor using the caliper method and a maximum absolute difference of 0.05 in hd-PS between paired 4F-PCC and historical plasma-treated patients.

### Statistical approach

We used SAS, version 9.4 (Cary, NC) for all analyses. We compared baseline characteristics in the matched cohort using standardized mean differences [19, 20]. Comparison of characteristics in unmatched cohorts is shown Supplemental Tables 2 and 3. We calculated cumulative incidence and incidence rates per 100 person-days for each outcome with associated 95% confidence intervals. We conducted Cox proportional hazards models to assess the association between VKA reversal strategy and outcomes at 7-, 14- and 45-days of follow-up, with additional adjustment for any residual differences in demographics, comorbidities, laboratory results and medications between groups using backward selection. Final variables included in each model based on backward selection are shown in Supplemental Table 4. As a sensitivity analysis, we conducted the same analyses on a matched cohort of 4F-PCC and contemporary plasma patients (which used the same matching procedure as for the main analyses) to assess for possible impact of temporal trends in outcomes or changes in measured confounders.

## Results

### Study cohorts and baseline characteristics

**Fig. 1:**
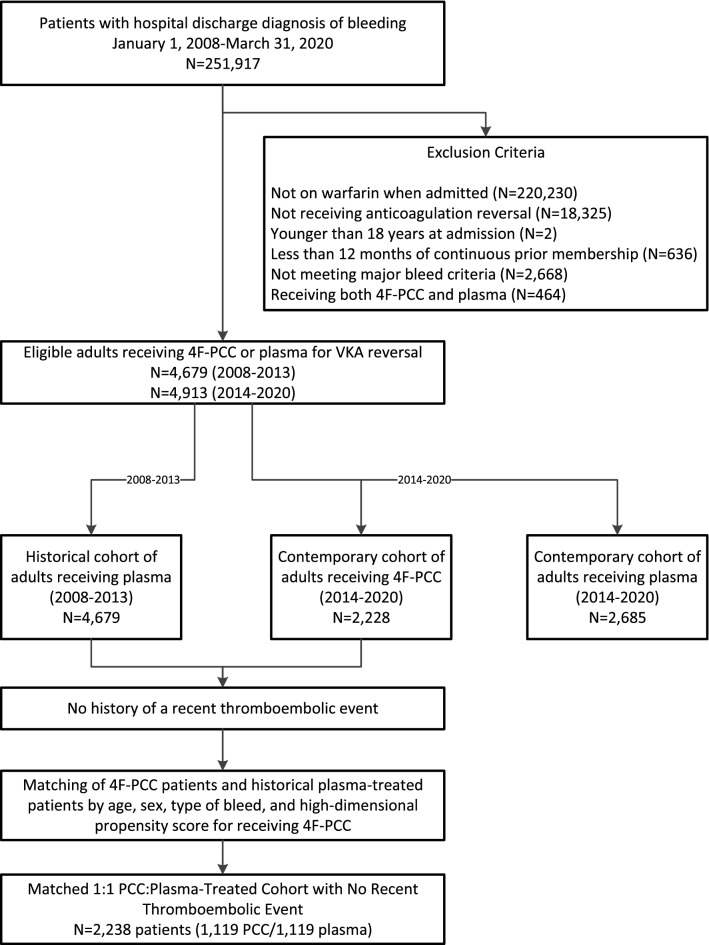
Identification of eligible adults treated with 4F-PCC or plasma for acute VKA reversal due to major bleeding

We identified 4679 eligible patients receiving plasma for VKA reversal between 2008 and 2013, and 2228 patients receiving 4F-PCC and 2685 patients receiving plasma between 2014 and 2020. After matching, we identified 1119 4F-PCC patients and 1119 historical plasma patients with no recent TEE (Fig. 1). In the matched cohort, compared with plasma-treated patients, those receiving 4F-PCC were more likely to be persons of color; receive warfarin for atrial fibrillation, remote prior venous thromboembolic disease, or valvular heart disease; receive a higher dose of oral vitamin K during the index hospitalization; have a higher prevalence of remote TEE (> 90 days before index date), heart failure, peripheral artery disease, prior hospitalized extracranial hemorrhage, hypercoagulable states, hypertension, dyslipidemia, diabetes and chronic liver disease; prior end-stage renal disease; and a higher pre-admission body mass index (Table 1).

**Table 1:** Baseline characteristics of matched adults with no recent history of thromboembolism treated with 4F-PCC or plasma for acute VKA reversal due to major bleeding

Variable	4F-PCC-treated patients n = 1119	Plasma-treated patients n = 1119	Standardized difference
Mean (SD) age, yr	77.0 (10.3)	76.4 (10.8)	0.05
Gender, N (%)			0.00
Men	609 (54.4)	609 (54.4)	
Women	510 (45.6)	510 (45.6)	
Race, N (%)			0.24
White	743 (66.4)	851 (76.1)	
Black	130 (11.6)	81 (7.2)	
Asian or Pacific Islander	181 (16.2)	145 (13.0)	
Other/Unknown	65 (5.8)	42 (3.8)	
Hispanic ethnicity, N (%)	186 (16.6)	166 (14.8)	0.05
Low educational attainment, N (%)	179 (16.0)	227 (20.3)	0.11
Low annual household income, N (%)	84 (7.5)	113 (10.1)	0.09
Indication for warfarin treatment, N (%)			
Atrial fibrillation	877 (78.4)	833 (74.4)	0.09
Venous thromboembolic disease	123 (11.0)	66 (5.9)	0.18
Valvular heart disease	321 (28.7)	235 (21.0)	0.18
Other/unknown	135 (12.1)	204 (18.2)	0.17
Type of index bleeding event, N (%)			0.08
Intracranial	757 (67.6)	757 (67.6)	
Gastrointestinal	350 (31.3)	339 (30.3)	
Other major extracranial	12 (1.1)	23 (2.1)	
Last INR value before VKA reversal			
Mean (SD)	2.7 (1.0)	2.7 (1.1)	0.05
Median (IQR)	2.5 (2.2–2.9)	2.5 (2.1–3.0)	
INR Category			0.09
< 2.0	165 (14.7)	174 (15.5)	
2.0 to < 4.0	829 (74.1)	777 (69.4)	
4.0 to < 6.0	94 (8.4)	146 (13.0)	
≥ 6.0	18 (1.6)	18 (1.6)	
Unknown	13 (1.2)	4 (0.4)	
In 4F-PCC-treated patients			
Median (SD) dose, units	2132 (1666–2598)		
In plasma-treated patients			
Median (IQR) units		2.0 (2.0–4.0)	
Oral vitamin K received, mg			
Median (IQR)	20.0 (10.0–20.0)	10.0 (5.0–20.0)	0.36
History of TEE, N (%)			0.12
None	828 (74.0)	884 (79.0)	
≤ 90 days	0 (0.0)	0 (0.0)	
> 90 days	291 (26.0)	235 (21.0)	
Medical history, N (%)			
Ischemic stroke	117 (10.5)	106 (9.5)	0.03
Acute coronary syndrome	129 (11.5)	106 (9.5)	0.07
Coronary revascularization	105 (9.4)	85 (7.6)	0.06
Heart failure	493 (44.1)	406 (36.3)	0.16
Peripheral artery disease	137 (12.2)	63 (5.6)	0.23
Intracranial hemorrhage	33 (2.9)	20 (1.8)	0.08
Hospitalized extracranial hemorrhage	75 (6.7)	49 (4.4)	0.10
Inherited coagulopathy	0 (0.0)	2 (0.2)	0.06
Hypercoagulable states	35 (3.1)	16 (1.4)	0.11
Hypertension	993 (88.7)	955 (85.3)	0.10
Dyslipidemia	978 (87.4)	915 (81.8)	0.16
Diabetes mellitus	507 (45.3)	346 (30.9)	0.30
Chronic liver disease	70 (6.3)	49 (4.4)	0.08
Chronic lung disease	412 (36.8)	407 (36.4)	0.01
Tobacco use, N (%)			0.07
None	533 (47.6)	560 (50.0)	
Former	548 (49.0)	510 (45.6)	
Current	38 (3.4)	49 (4.4)	
Baseline medication use, N (%)			
ACE inhibitor	301 (26.9)	447 (39.9)	0.28
Angiotensin II receptor blocker	205 (18.3)	156 (13.9)	0.12
Beta blocker	730 (65.2)	679 (60.7)	0.09
Calcium channel blocker	245 (21.9)	307 (27.4)	0.13
Diuretic	502 (44.9)	487 (43.5)	0.03
Aldosterone receptor antagonist	54 (4.8)	42 (3.8)	0.05
Alpha blocker	125 (11.2)	139 (12.4)	0.04
Statin	759 (67.8)	697 (62.3)	0.12
Non-statin lipid-lowering agent	31 (2.8)	74 (6.6)	0.18
Aspirin	34 (3.0)	15 (1.3)	0.12
Non-aspirin antiplatelet agent	41 (3.7)	41 (3.7)	0.00
Low molecular weight heparin	26 (2.3)	26 (2.3)	0.00
Non-steroidal anti-inflammatory drug	6 (0.5)	24 (2.1)	0.14
Systolic blood pressure, mmHg			
Mean (SD)	124.4 (17.6)	124.8 (17.9)	0.02
Diastolic blood pressure, mmHg			
Mean (SD)	66.7 (12.3)	68.6 (12.3)	0.15
Body mass index, kg/m^2^			
Mean (SD)	28.1 (6.4)	27.3 (6.1)	0.13
Estimated glomerular filtration rate (eGFR), ml/min/1.73 m^2^			
Mean (SD)	59.5 (22.1)	61.9 (21.2)	0.11
eGFR Category, N (%)			0.27
90–150 ml/min/1.73 m^2^	80 (7.1)	91 (8.1)	
60–89 ml/min/1.73 m^2^	415 (37.1)	421 (37.6)	
45–59 ml/min/1.73 m^2^	226 (20.2)	273 (24.4)	
30–44 ml/min/1.73 m^2^	157 (14.0)	146 (13.0)	
15–29 ml/min/1.73 m^2^	94 (8.4)	55 (4.9)	
< 15 ml/min/1.73 m^2^	15 (1.3)	12 (1.1)	
Chronic dialysis, N (%)	72 (6.4)	23 (2.1)	
Prior kidney transplant, N (%)	14 (1.3)	8 (0.7)	

### Clinical outcomes

During 45-day follow-up after acute VKA reversal, the overall risk of confirmed arterial or venous TEE after either 4F-PCC or plasma was 4.0% [95% confidence interval (CI) 3.3–4.9%], with 39 TEE (3.5%, 95% CI 2.5–4.7%) among 4F-PCC patients and 50 TEE (4.5%, 95% CI 3.3–5.9%) among historical plasma patients in the matched cohort (Table 2). The event rate (per 100 person-days) of TEE was 0.085 (95% CI 0.062–0.120) in 4F-PCC patients and 0.11 (95% CI 0.085–0.150) in historical plasma patients, with a corresponding rate ratio of 0.78 (95% CI 0.51–1.20) for 4F-PCC-treated patients.

**Table 2:** Distribution of thromboembolic events and all-cause death during the 45 days following treatment with 4F-PCC or plasma for acute VKA reversal in matched adults with major bleeding

Outcome	4F-PCC		Plasma	
	N	Rate per 100 person-days (95% CI)	N	Rate per 100 person-days (95% CI)
All thromboembolic events	39	0.085 (0.062–0.12)	50	0.11 (0.085–0.15)
Venous thromboembolism				
Deep venous thrombosis	17	0.037 (0.023–0.060)	15	0.034 (0.020–0.056)
Pulmonary embolism	6	0.013 (0.006–0.029)	5	0.011 (0.005–0.027)
Other venous thromboembolism	2	0.004 (0.001–0.017)	2	0.005 (0.001–0.018)
Arterial thromboembolism				
Acute myocardial infarction	1	0.002 (0.000–0.016)	5	0.011 (0.005–0.027)
Unstable angina	0	0.000 (0.000–0.000)	0	0.000 (0.000–0.000)
Ischemic stroke	11	0.024 (0.013–0.043)	22	0.049 (0.033–0.075)
Acute extremity embolism	0	0.000 (0.000–0.000)	1	0.002 (0.000–0.016)
Other arterial embolism	2	0.004 (0.001–0.017)	0	0.000 (0.000–0.000)
Death from any cause	144	0.32 (0.27–0.37)	206	0.46 (0.40–0.53)

We also identified 144 deaths among 4F-PCC patients and 206 deaths among historical plasma patients during 45-day follow-up in the matched cohort (Table 2). The event rate (per 100 person-days) of death was 0.32 (95% CI 0.27–0.37) in 4F-PCC patients and 0.46 (95% CI 0.40–0.53) in historical plasma patients, with a corresponding rate ratio of 0.70 (95% CI 0.57–0.85) for 4F-PCC-treated patients.

### Multivariable association of VKA reversal strategy and TEE

**Fig. 2:**
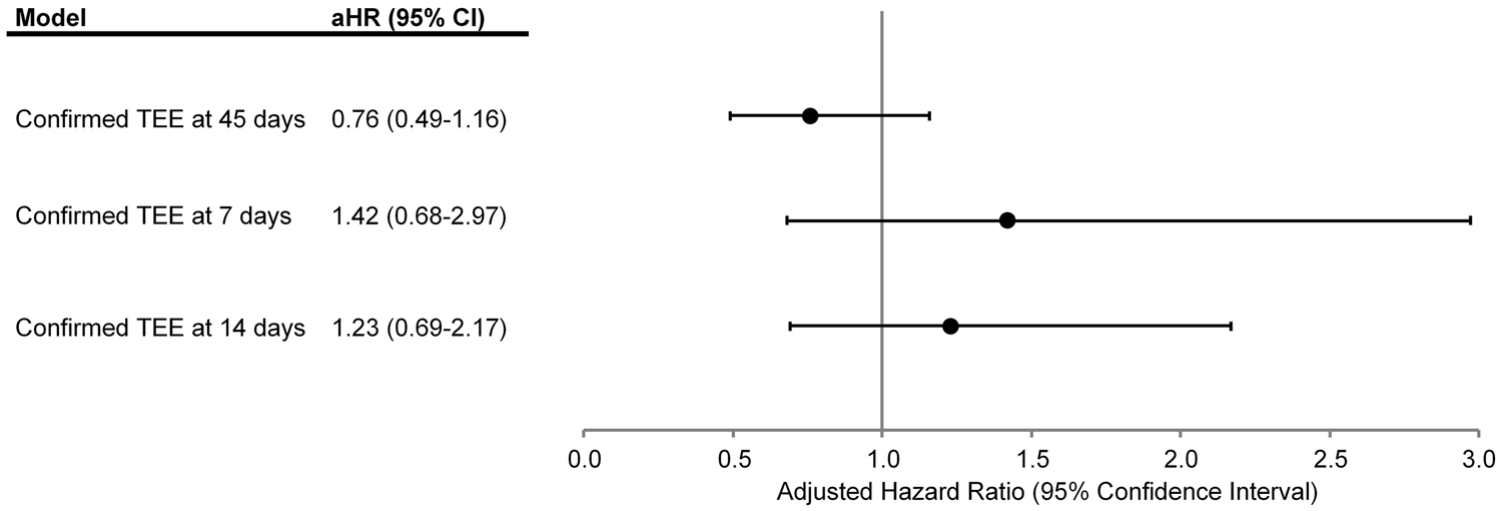
Multivariable association of 4F-PCC vs. plasma therapy with risk of thromboembolism at 7, 14 and 45 days post-treatment

Compared with historical patients receiving plasma, receipt of 4F-PCC was not significantly associated with an increased adjusted risk of TEE at 45 days post-treatment [adjusted hazard ratio (aHR) 0.76, 95% CI 0.49–1.16] (Fig. 2). Similarly, in sensitivity analyses, there were no significant adjusted differences in risk of TEE at 7- and 14-days post-treatment between patients receiving 4F-PCC compared with patients receiving plasma therapy (Fig. 2).

### Multivariable association of VKA reversal strategy and death

**Fig. 3:**
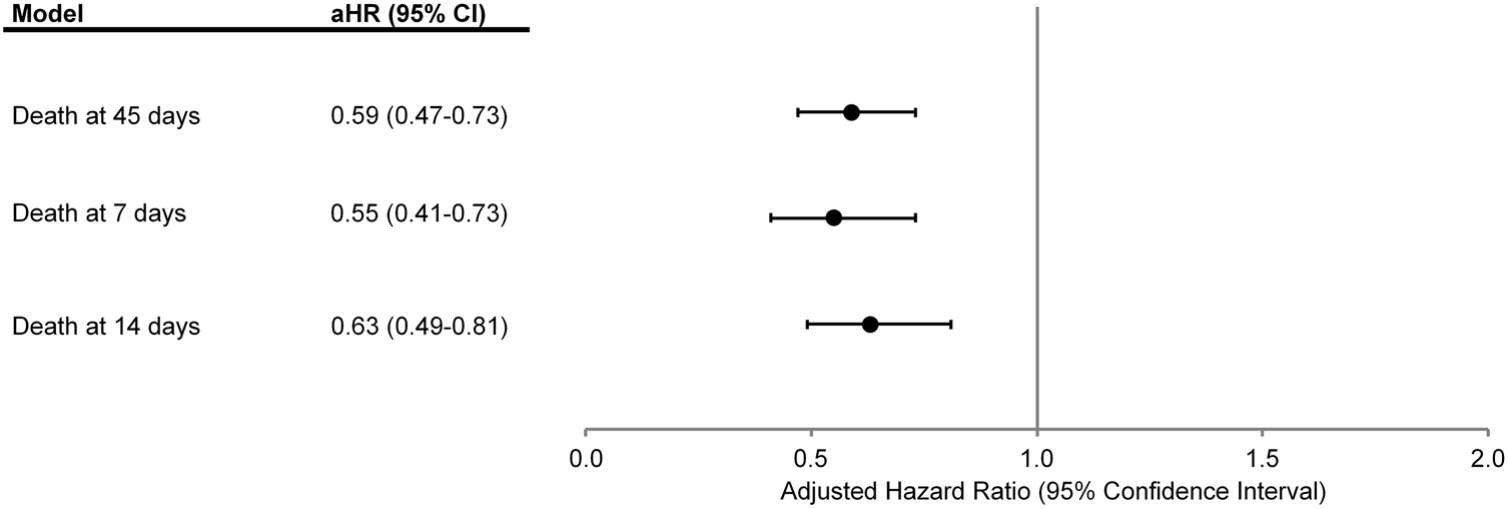
Multivariable association of 4F-PCC vs. plasma therapy with risk of death from any cause at 7, 14 and 45 days post-treatment

Compared with historical patients receiving plasma therapy, those who received 4F-PCC had a lower adjusted risk of all-cause death at 45 days (aHR 0.59, 95% CI 0.47–0.73) (Fig. 3). In sensitivity analyses, the favorable association of receipt of 4F-PCC compared with plasma therapy for all-cause death was also observed at 7- and 14-days post-treatment (Fig. 3).

### Additional sensitivity analyses

In a sensitivity analysis within a matched cohort of 667 patients receiving 4F-PCC and 667 contemporary plasma-treated patients, there was no significant difference in the multivariable risk of TEE at 45 days post-treatment for those receiving 4F-PCC compared with plasma (aHR 0.92, 95% CI 0.55–1.54). In a similar sensitivity analysis, we found no significant difference in the multivariable risk of all-cause death in those receiving 4F-PCC compared with contemporary plasma-treated patients (aHR 0.80, 95% CI 0.59–1.10).

## Discussion

In this large, diverse, matched cohort study of adults requiring acute VKA reversal for major bleeding in typical practice settings, there was no significant difference between those receiving 4F-PCC compared with plasma in the short-term risk of post-treatment TEE despite those receiving 4F-PCC having a higher prevalence of TEE risk factors. Of note, we observed a lower adjusted difference in the risk of all-cause death at 45 days post-treatment in those receiving 4F-PCC compared with a historical cohort of patients who received plasma.

Human plasma has been a standard of care option for VKA reversal, yet its efficacy has not been reliably determined in randomized controlled trials, along with having limitations such as restricted international availability, infection-related risks, the requirement for ABO blood typing and preparation time before infusion, and transfusion-related complications [21–23]. The emergence of PCC, including the 4F-PCC (Factor II, VII, IX and X), examined in this study, has important advantages over plasma, including avoiding the need for any cross-matching or material thawing, minimal infection-related risks, no risk of transfusion-associated acute lung injury, short infusion time, more rapid INR reversal, and more widespread availability albeit at a significantly higher per unit cost [21–23]. Importantly, however, only limited data exist about comparative safety outcomes between 4F-PCC and plasma, and specifically potential differences in the risk of TEE off VKA therapy among patients prone to TEE. Using combined data from two randomized trials, Milling et al. examined the incidence of TEE and death among 191 patients receiving 4F-PCC and 197 receiving plasma for major bleeding [24, 25]. The overall 45-day risk of any TEE was 7.2% (95% CI 4.9–10.3%), with 14 (7.3%) 4F-PCC and 14 (7.1%) plasma-treated patients experiencing one or more TEE (risk difference 0.2%, 95% CI − 5.5–6.0%) [24]. In addition, the overall 45-day risk of all-cause death was 5.9% (95% CI 3.8–8.8%), affecting 13 (6.8%) 4F-PCC and 13 (6.6%) plasma-treated patients [25]. However, both analyses had very limited precision and included only selected trial participants. Subsequent systematic reviews and meta-analyses comparing outcomes of 4F-PCC or plasma involving clinical trials and observational studies noted multiple limitations of analyzed studies, including limited sample sizes, varying lengths of follow-up, different ascertainment methods and definitions of adverse outcomes, limited adjustment for confounding, and variable study quality [26–29]. Our findings materially extend and clarify the short-term absolute risk of TEE after acute VKA reversal and potential differences with the use of 4F-PCC versus plasma in a substantially larger and contemporary population. Our finding of lower adjusted all-cause mortality associated with receipt of 4F-PCC compared with a historical plasma-treated cohort is consistent with some but not all studies, [26–29] and we note that in a sensitivity analysis between 4F-PCC and contemporary plasma-treated patients, there was no significant difference in adjusted survival.

Our study has several important strengths. To our knowledge, this is the largest evaluation of potential adverse drug reactions associated with the use of 4F-PCC compared with plasma following VKA reversal in the setting of major bleeding, which provides precise and generalizable estimates of the 45-day risk of post-treatment TEE. To reduce the effects of potential selection bias and confounding, we employed matching on individual-level confounders as well as on a high-dimensional, well-discriminating propensity score for receiving 4F-PCC, and statistical adjustment of any remaining differences in measured patient characteristics. Our matched multicenter cohort included diverse patients across the spectrum of age, sex, race/ethnicity and socioeconomic status. We also confirmed the occurrence and timing of post-treatment TEE through physician adjudication of medical records using standardized diagnostic criteria with adjudicators who were blinded to VKA reversal strategy.

There were also several study limitations. We did not use the exact International Society on Thrombosis and Haemostasis (ISTH) criteria for major bleeding due to concerns about complete data availability, but our definition was highly consistent with the ISTH approach. For the least biased evaluation, we compared patients receiving 4F-PCC with a recent historical matched cohort of patients receiving plasma therapy, which is susceptible to bias if there were any material temporal trends in other management strategies that may influence post-VKA reversal outcomes. However, in our sensitivity analysis of TEE involving matched contemporary plasma-treated patients, the results were similar to the main analyses. Our pre-specified target population was patients with no recent history of TEE, and we were unable to evaluate potential differences in outcomes among those with a recent TEE due to the limited number of patients. We were also unable to address potential differences in outcomes for a strategy of combined 4F-PCC and plasma therapy versus each approach alone, or the use of other PCC formulations. We were also unable to distinguish between type 2 acute coronary syndrome vs. acute myocardial infarction secondary to coronary thrombosis. As an observational study of treatment-associated outcomes, we cannot rule out the effects of unmeasured confounding or treatment selection bias. However, while we achieved excellent matching between groups, those receiving 4F-PCC had a residual higher prevalence of TEE risk factors compared with matched plasma-treated patients, which would bias towards worse rather than better outcomes in those receiving 4F-PCC. While our patients were obtained from a sociodemographically diverse source population of nearly 10 million persons with comprehensive inpatient and outpatient electronic health record information, our findings may not be completely generalizable to uninsured patients or other geographic practice settings.

In conclusion, we observed that a TEE occurred in approximately 1 in 25 patients after acute VKA reversal for major bleeding, but there was no significant adjusted difference in TEE risk with receipt of 4F-PCC compared with plasma therapy. Furthermore, adjusted risk of all-cause death at 45 days post-treatment was lower in patients receiving 4F-PCC compared with a matched historical cohort receiving plasma therapy. Our study provides reassurance about the safety of 4F-PCC for the management of VKA-associated major bleeding.

## Supplementary Information

Below is the link to the electronic supplementary material.

Supplementary file1 (DOCX 43 KB)

## References

[1] Sarode R, Milling TJ, Refaai MA, Mangione A, Schneider A, Durn BL (2013). Efficacy and safety of a 4-factor prothrombin complex concentrate in patients on vitamin K antagonists presenting with major bleeding: a randomized, plasma-controlled, phase IIIb study. Circulation.

[2] Goldstein JN, Refaai MA, Milling TJ, Lewis B, Goldberg-Alberts R, Hug BA (2015). Four-factor prothrombin complex concentrate versus plasma for rapid vitamin K antagonist reversal in patients needing urgent surgical or invasive interventions: a phase 3b, open-label, non-inferiority, randomised trial. Lancet.

[3] Endres K, St Bernard R, Chin-Yee I, Hsia C, Lazo-Langner A (2020). Efficacy and safety of four-factor prothrombin complex concentrate fixed, weight-based dosing for reversal of warfarin anticoagulation. Hematology.

[4] Jones GM, Erdman MJ, Smetana KS, Mohrien KM, Vandigo JE, Elijovich L (2016). 3-Factor versus 4-factor prothrombin complex concentrate for warfarin reversal in severe bleeding: a multicenter, retrospective, propensity-matched pilot study. J Thromb Thrombolysis.

[5] Kuroski JE, Young S (2017). Comparison of the safety and efficacy between 3-factor and 4-factor prothrombin complex concentrates for the reversal of warfarin. Am J Emerg Med.

[6] DeLoughery EP, DeLoughery TG (2017). Use of three procoagulants in improving bleeding outcomes in the warfarin patient with intracranial hemorrhage. Blood Coagul Fibrinolysis.

[7] Margraf DJ, Seaburg S, Beilman GJ, Wolfson J, Gipson JC, Chapman SA (2020). Propensity score adjusted comparison of three-factor versus four-factor prothrombin complex concentrate for emergent warfarin reversal: a retrospective cohort study. BMC Emerg Med.

[8] Rowe AS, Dietrich SK, Phillips JW, Foster KE, Canter JR (2018). Activated prothrombin complex concentrate versus 4-factor prothrombin complex concentrate for vitamin K-antagonist reversal. Crit Care Med.

[9] Fischer D, Sorensen J, Fontaine GV (2018). Three-factor versus four-factor prothrombin complex concentrate for the emergent management of warfarin-associated intracranial hemorrhage. Neurocrit Care.

[10] Koebnick C, Langer-Gould AM, Gould MK, Chao CR, Iyer RL, Smith N (2012). Sociodemographic characteristics of members of a large, integrated health care system: comparison with US Census Bureau data. Perm J.

[11] Gordon NP (2020) Similarity of adult Kaiser Permanente members to the adult population in Kaiser Permanente’s Northern California service area: Comparisons based on the 2017/2018 cycle of the California Health Interview Survey. Report prepared for the Kaiser Permanente Division of Research, Oakland, CA, November 8, 2020. Available at https://divisionofresearch.kaiserpermanente.org/projects/memberhealthsurvey/SiteCollectionDocuments/compare_kp_ncal_chis2017-18.pdf

[12] Ross TR, Ng D, Brown JS, Pardee R, Hornbrook MC, Hart G (2014). The HMO research network virtual data warehouse: a public data model to support collaboration. EGEMS.

[13] Magid DJ, Gurwitz JH, Rumsfeld JS, Go AS (2008). Creating a research data network for cardiovascular disease: the CVRN. Expert Rev Cardiovasc Ther.

[14] Arellano MG, Petersen GR, Petitti DB, Smith RE (1984). The california automated mortality linkage system (CAMLIS). Am J Public Health.

[15] Go AS, Chertow GM, Fan D, McCulloch CE, Hsu CY (2004). Chronic kidney disease and the risks of death, cardiovascular events, and hospitalization. N Engl J Med.

[16] Go AS, Lee WY, Yang J, Lo JC, Gurwitz JH (2006). Statin therapy and risks for death and hospitalization in chronic heart failure. JAMA.

[17] Goldberg RJ, Gurwitz JH, Saczynski JS, Hsu G, McManus DD, Magid DJ (2013). Comparison of medication practices in patients with heart failure and preserved versus those with reduced ejection fraction [from the cardiovascular research network (CVRN)]. Am J Cardiol.

[18] Schneeweiss S, Rassen JA, Glynn RJ, Avorn J, Mogun H, Brookhart MA (2009). High-dimensional propensity score adjustment in studies of treatment effects using health care claims data. Epidemiology.

[19] Austin PC (2008). A critical appraisal of propensity-score matching in the medical literature between 1996 and 2003. Stat Med.

[20] Austin PC (2009). Using the standardized difference to compare the prevalence of a binary variable between two groups in observational research. Commun Stat Simul Comput.

[21] Ortel TL, Neumann I, Ageno W, Beyth R, Clark NP, Cuker A (2020). American society of hematology 2020 guidelines for management of venous thromboembolism: treatment of deep vein thrombosis and pulmonary embolism. Blood Adv.

[22] Ageno W, Gallus AS, Wittkowsky A, Crowther M, Hylek EM, Palareti G (2012). Oral anticoagulant therapy: antithrombotic therapy and prevention of thrombosis, 9th ed: American college of chest physicians evidence-based clinical practice guidelines. Chest.

[23] Food and Drug Administration. Fatalities reported to FDA following blood collection and transfusion annual summary for FY2019. Available at https://www.fda.gov/media/147628/download. Accessed on 13 Nov 2021

[24] Milling TJ, Refaai MA, Goldstein JN, Schneider A, Omert L, Harman A (2016). Thromboembolic events after vitamin K antagonist reversal with 4-factor prothrombin complex concentrate: exploratory analyses of two randomized, plasma-controlled studies. Ann Emerg Med.

[25] Milling TJ, Refaai MA, Sarode R, Lewis B, Mangione A, Durn BL (2016). Safety of a four-factor prothrombin complex concentrate versus plasma for vitamin K antagonist reversal: an integrated analysis of two phase IIIb clinical trials. Acad Emerg Med.

[26] van den Brink DP, Wirtz MR, Neto AS, Schochl H, Viersen V, Binnekade J (2020). Effectiveness of prothrombin complex concentrate for the treatment of bleeding: a systematic review and meta-analysis. J Thromb Haemost.

[27] Pan R, Cheng J, Lai K, Huang Q, Wu H, Tang Y (2019). Efficacy and safety of prothrombin complex concentrate for vitamin K antagonist-associated intracranial hemorrhage: a systematic review and meta-analysis. Neurol Sci.

[28] Brekelmans MPA, Ginkel KV, Daams JG, Hutten BA, Middeldorp S, Coppens M (2017). Benefits and harms of 4-factor prothrombin complex concentrate for reversal of vitamin K antagonist associated bleeding: a systematic review and meta-analysis. J Thromb Thrombolysis.

[29] Johansen M, Wikkelso A, Lunde J, Wetterslev J, Afshari A (2015). Prothrombin complex concentrate for reversal of vitamin K antagonist treatment in bleeding and non-bleeding patients. Cochrane Database Syst Rev.

