# Supplementary material for: Thromboembolism after treatment with 4-factor prothrombin complex concentrate or plasma for warfarin-related bleeding
Source: J Thromb Thrombolysis. 2022 Aug 19;54(3):470–9. doi: 10.1007/s11239-022-02695-5 (PMC9553785; doi:10.1007/s11239-022-02695-5)
Supplement: Supplementary file 1 — Supplementary file1 (DOCX 43 KB) [file 11239_2022_2695_MOESM1_ESM.docx]

**Supplementary Materials**

**Thromboembolism After Treatment with 4-Factor Prothrombin Complex Concentrate or Plasma for Warfarin-Related Bleeding**

*Short Title: Thromboembolism After 4F-PCC or Plasma Therapy*

Alan S. Go, MD,^1,2,3,4^ Thomas K. Leong, MPH,^1^ Sue Hee Sung, MPH,^1^ Rong Wei, MA,^5^

Teresa N. Harrison, SM,^5^ Nigel Gupta, MD,^6^ Nicole Baker, MPH,^7^ Brahm Goldstein, MD, MCR,^7^ Quazi Ataher, PhD, MBBS, MHS,^7^ Matthew D. Solomon, MD, PhD,^1,8^ and Kristi Reynolds, PhD, MPH^2,5^ for the REVERSAL Study

^1^Division of Research, Kaiser Permanente Northern California, Oakland, CA; ^2^Department of Health Systems Science, Kaiser Permanente Bernard J. Tyson School of Medicine, Pasadena, CA; ^3^Departments of Epidemiology, Biostatistics and Medicine, University of California, San Francisco, San Francisco, CA; ^4^Department of Medicine, Stanford University, Palo Alto, CA; ^5^Department of Research and Evaluation, Kaiser Permanente Southern California, Pasadena, CA; ^6^Department of Cardiac Electrophysiology, Southern CA Permanente Medical Group, Los Angeles, CA, ^7^Clinical Epidemiology, CSL Behring, King of Prussia, PA, ^8^Department of Cardiology, Kaiser Permanente Oakland Medical Center, Oakland, CA

Address for Correspondence: Alan S. Go, M.D.

Division of Research

Kaiser Permanente Northern California

2000 Broadway, Oakland, CA 94612

Email: alan.s.go@kp.org

**Supplemental Table 1. Criteria used for each study outcome.**

| **Outcome** | **Definition** |
| --- | --- |
| Ischemic stroke | Thrombus related to neurologic deficit with duration >24 hours and with evidence of acute brain infarction via MRI or CT scan. |
| Transient ischemic attack | Thrombus related to neurologic deficit with duration ≤24 hours. |
| Venous thromboembolism | Thrombus in a deep vein, not including superficial vein thrombosis or thrombophlebitis. Must be validated by diagnostic studies such as ultrasound, venography, CT or MRI. May be detected incidentally as part of investigation of other clinical complaints. |
| Pulmonary embolism | Thrombus in a deep vein causing an embolism in a pulmonary artery. Must be validated by diagnostic studies such as venography, CT or pathology/autopsy. |
| Other arterial thromboembolic event | Peripheral arterial embolus. Must specify whether occlusion was documented by angiography and, if so, whether or not atherosclerotic plaque was found in the affected arteries. Must specify the site of the peripheral embolus. |
| Acute myocardial infarction | Clinical encounter meeting one or more of the following criteria:  - Detection of troponin with at least one value above the 99th percentile of the upper reference limit (URL) with at least one of the following:     - Symptoms of acute ischemia     - ECG changes indicative of new ischemia     - Development of pathological Q waves in the ECG     - Imaging evidence of new loss of viable myocardium or new regional wall motion abnormality  - Sudden, unexpected cardiac death, involving cardiac arrest and accompanied by ST elevation or new LBBB, and/or evidence of fresh thrombus by coronary angiography and/or at autopsy.  - Pathological findings of an acute myocardial infarction. |
| Unstable angina | New onset of ischemic symptoms, intensification of previous ischemic symptoms, or recurrence of ischemic symptoms within 4-6 weeks after an acute myocardial infarction. Excludes episodes meeting the definition of acute myocardial infarction. |

Supplemental Table 2. Baseline characteristics of all adults treated with 4F-PCC (2013-2020) or plasma (2008-2012) for acute VKA reversal due to major bleeding.

| Variable | 4F-PCC-treated patients  n = 2228 | Plasma-treated patients  n = 4679 | Standardized difference |
| --- | --- | --- | --- |
| Mean (SD) age, yr | 76.3 (11.0) | 75.4 (11.3) | 0.08 |
| Gender, N (%) |  |  | 0.01 |
| Men | 1238 (55.6) | 2615 (55.9) |  |
| Women | 990 (44.4) | 2064 (44.1) |  |
| Race, N (%) |  |  | **0.22** |
| White | 1465 (65.8) | 3455 (73.8) |  |
| Black | 241 (10.8) | 505 (10.8) |  |
| Asian or Pacific Islander | 378 (17.0) | 548 (11.7) |  |
| Other/Unknown | 144 (6.5) | 171 (3.7) |  |
| Hispanic ethnicity, N (%) | 385 (17.3) | 720 (15.4) | 0.05 |
| Low educational attainment, N (%) | 357 (16.0) | 1073 (22.9) | 0.18 |
| Low annual household income, N (%) | 140 (6.3) | 576 (12.3) | **0.21** |
| Indication for warfarin treatment, N (%) |  |  |  |
| Atrial fibrillation | 1683 (75.5) | 3283 (70.2) | 0.12 |
| Venous thromboembolic disease | 301 (13.5) | 575 (12.3) | 0.04 |
| Valvular heart disease | 554 (24.9) | 1146 (24.5) | 0.01 |
| Other/unknown | 295 (13.2) | 795 (17.0) | 0.10 |
| Type of index bleeding event, N (%) |  |  | **0.86** |
| Intracranial | 1732 (77.7) | 1813 (38.7) |  |
| Gastrointestinal | 469 (21.1) | 2648 (56.6) |  |
| Other major extracranial | 27 (1.2) | 218 (4.7) |  |
| Last INR value before VKA reversal |  |  |  |
| Mean (SD) | 2.7 (1.0) | 2.8 (1.2) | 0.11 |
| Median (IQR) | 2.5 (2.2-2.9) | 2.6 (2.1-3.1) |  |
| INR Category |  |  | **0.22** |
| < 2.0 | 326 (14.6) | 799 (17.1) |  |
| 2.0 to <4.0 | 1636 (73.4) | 3104 (66.3) |  |
| 4.0 to <6.0 | 200 (9.0) | 606 (13.0) |  |
| ≥6.0 | 33 (1.5) | 118 (2.5) |  |
| Unknown | 33 (1.5) | 52 (1.1) |  |
| In Kcentra-treated patients |  |  |  |
| Median (SD) dose, units | 2128 (1665-2560) |  |  |
| In plasma-treated patients |  |  |  |
| Median (IQR) units |  | 3.0 (2.0-4.0) |  |
| Oral vitamin K received, mg |  |  |  |
| Median (IQR) | 20.0 (10.0-25.0) | 10.0 (5.0-15.0) | **0.62** |
| History of TEE, N (%) |  |  | 0.08 |
| None | 1560 (70.0) | 3123 (66.7) |  |
| ≤90 days | 169 (7.6) | 485 (10.4) |  |
| >90 days | 499 (22.4) | 1071 (22.9) |  |
| Medical history, N (%) |  |  |  |
| Ischemic stroke | 276 (12.4) | 579 (12.4) | 0.00 |
| Acute coronary syndrome | 223 (10.0) | 728 (15.6) | 0.17 |
| Coronary revascularization | 166 (7.5) | 564 (12.1) | 0.16 |
| Heart failure | 859 (38.6) | 1933 (41.3) | 0.06 |
| Peripheral artery disease | 256 (11.5) | 447 (9.6) | 0.06 |
| Intracranial hemorrhage | 84 (3.8) | 95 (2.0) | 0.10 |
| Hospitalized extracranial hemorrhage | 119 (5.3) | 468 (10.0) | 0.18 |
| Inherited coagulopathy | 1 (0.0) | 7 (0.1) | 0.03 |
| Hypercoagulable states | 77 (3.5) | 86 (1.8) | 0.10 |
| Hypertension | 1929 (86.6) | 4089 (87.4) | 0.02 |
| Dyslipidemia | 1935 (86.8) | 3927 (83.9) | 0.08 |
| Diabetes mellitus | 959 (43.0) | 1788 (38.2) | 0.10 |
| Chronic liver disease | 157 (7.0) | 227 (4.9) | 0.09 |
| Chronic lung disease | 791 (35.5) | 1781 (38.1) | 0.05 |
| Tobacco use, N (%) |  |  | 0.09 |
| None | 1090 (48.9) | 2103 (44.9) |  |
| Former | 1048 (47.0) | 2352 (50.3) |  |
| Current | 90 (4.0) | 224 (4.8) |  |
| Baseline medication use, N (%) |  |  |  |
| ACE inhibitor | 624 (28.0) | 1818 (38.9) | **0.23** |
| Angiotensin II receptor blocker | 408 (18.3) | 737 (15.8) | 0.07 |
| Beta blocker | 1384 (62.1) | 2873 (61.4) | 0.01 |
| Calcium channel blocker | 476 (21.4) | 1397 (29.9) | **0.20** |
| Diuretic | 910 (40.8) | 2286 (48.9) | 0.16 |
| Aldosterone receptor antagonist | 103 (4.6) | 227 (4.9) | 0.01 |
| Alpha ARA | 229 (10.3) | 655 (14.0) | 0.11 |
| Statin | 1459 (65.5) | 2965 (63.4) | 0.04 |
| Non-statin lipid-lowering agent | 62 (2.8) | 301 (6.4) | 0.17 |
| Aspirin | 76 (3.4) | 116 (2.5) | 0.06 |
| Non-aspirin antiplatelet agent | 92 (4.1) | 237 (5.1) | 0.04 |
| Low molecular weight heparin | 81 (3.6) | 221 (4.7) | 0.05 |
| NSAID | 18 (0.8) | 98 (2.1) | 0.11 |
| Systolic blood pressure, mmHg |  |  |  |
| Mean (SD) | 125.7 (17.9) | 122.6 (18.3) | 0.17 |
| Diastolic blood pressure, mmHg |  |  |  |
| Mean (SD) | 67.9 (12.0) | 66.8 (12.1) | 0.09 |
| Body mass index, kg/m^2^ |  |  |  |
| Mean (SD) | 28.3 (6.7) | 28.0 (6.5) | 0.04 |
| Estimated glomerular filtration rate (eGFR), ml/min/1.73 m^2^ |  |  |  |
| Mean (SD) | 61.9 (22.0) | 59.5 (22.6) | 0.11 |
| eGFR Category, N (%) |  |  | **0.20** |
| 90-150 ml/min/1.73 m^2^ | 193 (8.7) | 363 (7.8) |  |
| 60-89 ml/min/1.73 m^2^ | 871 (39.1) | 1582 (33.8) |  |
| 45-59 ml/min/1.73 m^2^ | 456 (20.5) | 1010 (21.6) |  |
| 30-44 ml/min/1.73 m^2^ | 279 (12.5) | 756 (16.2) |  |
| 15-29 ml/min/1.73 m^2^ | 150 (6.7) | 333 (7.1) |  |
| <15 ml/min/1.73 m^2^ | 21 (0.9) | 62 (1.3) |  |
| Chronic dialysis, N (%) | 115 (5.2) | 154 (3.3) |  |
| Prior kidney transplant, N (%) | 24 (1.1) | 49 (1.0) |  |

Supplemental Table 3. Baseline characteristics of all adults treated with 4F-PCC (2013-2020) or plasma (2013-2020) for acute VKA reversal due to major bleeding.

| Variable | 4F-PCC-treated patients  n = 2228 | Plasma-treated patients  n = 2685 | Standardized difference |
| --- | --- | --- | --- |
| Mean (SD) age, yr | 76.3 (11.0) | 76.0 (11.2) | 0.02 |
| Gender, N (%) |  |  | 0.06 |
| Men | 1238 (55.6) | 1568 (58.4) |  |
| Women | 990 (44.4) | 1117 (41.6) |  |
| Race, N (%) |  |  | 0.11 |
| White | 1465 (65.8) | 1819 (67.7) |  |
| Black | 241 (10.8) | 344 (12.8) |  |
| Asian or Pacific Islander | 378 (17.0) | 365 (13.6) |  |
| Other/Unknown | 144 (6.5) | 157 (5.8) |  |
| Hispanic ethnicity, N (%) | 385 (17.3) | 449 (16.7) | 0.01 |
| Low educational attainment, N (%) | 357 (16.0) | 521 (19.4) | 0.09 |
| Low annual household income, N (%) | 140 (6.3) | 251 (9.3) | 0.11 |
| Indication for warfarin treatment, N (%) |  |  |  |
| Atrial fibrillation | 1683 (75.5) | 1935 (72.1) | 0.08 |
| Venous thromboembolic disease | 301 (13.5) | 389 (14.5) | 0.03 |
| Valvular heart disease | 554 (24.9) | 732 (27.3) | 0.05 |
| Other/unknown | 295 (13.2) | 406 (15.1) | 0.05 |
| Type of index bleeding event, N (%) |  |  | **1.22** |
| Intracranial | 1732 (77.7) | 695 (25.9) |  |
| Gastrointestinal | 469 (21.1) | 1861 (69.3) |  |
| Other major extracranial | 27 (1.2) | 129 (4.8) |  |
| Last INR value before VKA reversal |  |  |  |
| Mean (SD) | 2.7 (1.0) | 2.7 (1.1) | 0.07 |
| Median (IQR) | 2.5 (2.2-2.9) | 2.6 (2.1-3.0) |  |
| INR Category |  |  | 0.16 |
| < 2.0 | 326 (14.6) | 448 (16.7) |  |
| 2.0 to <4.0 | 1636 (73.4) | 1842 (68.6) |  |
| 4.0 to <6.0 | 200 (9.0) | 306 (11.4) |  |
| ≥6.0 | 33 (1.5) | 60 (2.2) |  |
| Unknown | 33 (1.5) | 29 (1.1) |  |
| In Kcentra-treated patients |  |  |  |
| Median (SD) dose, units | 2128 (1665-2560) |  |  |
| In plasma-treated patients |  |  |  |
| Median (IQR) units |  | 2.0 (2.0-4.0) |  |
| Oral vitamin K received, mg |  |  |  |
| Median (IQR) | 20.0 (10.0-25.0) | 10.0 (5.0-10.0) | **0.82** |
| History of TEE, N (%) |  |  | 0.08 |
| None | 1560 (70.0) | 1786 (66.5) |  |
| ≤90 days | 169 (7.6) | 275 (10.2) |  |
| >90 days | 499 (22.4) | 624 (23.2) |  |
| Medical history, N (%) |  |  |  |
| Ischemic stroke | 276 (12.4) | 324 (12.1) | 0.01 |
| Acute coronary syndrome | 223 (10.0) | 400 (14.9) | 0.15 |
| Coronary revascularization | 166 (7.5) | 316 (11.8) | 0.15 |
| Heart failure | 859 (38.6) | 1141 (42.5) | 0.08 |
| Peripheral artery disease | 256 (11.5) | 361 (13.4) | 0.06 |
| Intracranial hemorrhage | 84 (3.8) | 112 (4.2) | 0.02 |
| Hospitalized extracranial hemorrhage | 119 (5.3) | 327 (12.2) | **0.24** |
| Inherited coagulopathy | 1 (0.0) | 1 (0.0) | 0.00 |
| Hypercoagulable states | 77 (3.5) | 89 (3.3) | 0.01 |
| Hypertension | 1929 (86.6) | 2340 (87.2) | 0.02 |
| Dyslipidemia | 1935 (86.8) | 2342 (87.2) | 0.01 |
| Diabetes mellitus | 959 (43.0) | 1182 (44.0) | 0.02 |
| Chronic liver disease | 157 (7.0) | 200 (7.4) | 0.02 |
| Chronic lung disease | 791 (35.5) | 1084 (40.4) | 0.10 |
| Tobacco use, N (%) |  |  | 0.12 |
| None | 1090 (48.9) | 1160 (43.2) |  |
| Former | 1048 (47.0) | 1400 (52.1) |  |
| Current | 90 (4.0) | 125 (4.7) |  |
| Baseline medication use, N (%) |  |  |  |
| ACE inhibitor | 624 (28.0) | 828 (30.8) | 0.06 |
| Angiotensin II receptor blocker | 408 (18.3) | 479 (17.8) | 0.01 |
| Beta blocker | 1384 (62.1) | 1660 (61.8) | 0.01 |
| Calcium channel blocker | 476 (21.4) | 682 (25.4) | 0.10 |
| Diuretic | 910 (40.8) | 1239 (46.1) | 0.11 |
| Aldosterone receptor antagonist | 103 (4.6) | 139 (5.2) | 0.03 |
| Alpha ARA | 229 (10.3) | 320 (11.9) | 0.05 |
| Statin | 1459 (65.5) | 1736 (64.7) | 0.02 |
| Non-statin lipid-lowering agent | 62 (2.8) | 87 (3.2) | 0.03 |
| Aspirin | 76 (3.4) | 105 (3.9) | 0.03 |
| Non-aspirin antiplatelet agent | 92 (4.1) | 157 (5.8) | 0.08 |
| Low molecular weight heparin | 81 (3.6) | 203 (7.6) | 0.17 |
| NSAID | 18 (0.8) | 40 (1.5) | 0.06 |
| Systolic blood pressure, mmHg |  |  |  |
| Mean (SD) | 125.7 (17.9) | 122.1 (18.2) | **0.20** |
| Diastolic blood pressure, mmHg |  |  |  |
| Mean (SD) | 67.9 (12.0) | 65.7 (12.4) | 0.18 |
| Body mass index, kg/m^2^ |  |  |  |
| Mean (SD) | 28.3 (6.7) | 28.6 (7.0) | 0.05 |
| Estimated glomerular filtration rate (eGFR), ml/min/1.73 m^2^ |  |  |  |
| Mean (SD) | 61.9 (22.0) | 59.1 (22.8) | 0.13 |
| eGFR Category, N (%) |  |  | 0.17 |
| 90-150 ml/min/1.73 m^2^ | 193 (8.7) | 207 (7.7) |  |
| 60-89 ml/min/1.73 m^2^ | 871 (39.1) | 899 (33.5) |  |
| 45-59 ml/min/1.73 m^2^ | 456 (20.5) | 569 (21.2) |  |
| 30-44 ml/min/1.73 m^2^ | 279 (12.5) | 450 (16.8) |  |
| 15-29 ml/min/1.73 m^2^ | 150 (6.7) | 193 (7.2) |  |
| <15 ml/min/1.73 m^2^ | 21 (0.9) | 39 (1.5) |  |
| Chronic dialysis, N (%) | 115 (5.2) | 177 (6.6) |  |
| Prior kidney transplant, N (%) | 24 (1.1) | 35 (1.3) |  |

**Supplemental Table 4. Final covariates retained using a backward selection procedure for each multivariable regression model.**

| **4F-PCC** | **Plasma** | **Outcome** | **Final model covariates** |
| --- | --- | --- | --- |
| 2013-2020 | 2008-2012 | Confirmed TEE, 7 days | Gender  Time-varying systolic BP  Time-varying diastolic BP  Index age  Baseline history of CABG  Time-varying history of dyslipidemia  Time-varying prescription of ARBs  Receipt of injectable vitamin K  Receipt of procedure to stop bleeding |
| 2013-2020 | 2008-2012 | Confirmed TEE, 14 days | Time-varying systolic BP  Time-varying diastolic BP  Index age  Baseline history of CABG  Receipt of injectable vitamin K  Receipt of procedure to stop bleeding |
| 2013-2020 | 2008-2012 | Confirmed TEE, 45 days | Gender  Time-varying diastolic BP  Index age  Time-varying history of atrial flutter or fibrillation  Baseline history of acute MI  Time-varying prescription of alpha ARAs  Receipt of 2 or more units of RBCs  Receipt of procedure to stop bleeding |
| 2013-2020 | 2008-2012 | Death, 7 days | Baseline history of VTE  Time-varying history of diabetes  Time-varying prescription of statins  Time-varying prescription of non-statin lipid therapy  Receipt of oral vitamin K  Receipt of 2 or more units of RBCs |
| 2013-2020 | 2008-2012 | Death, 14 days | Time-varying systolic BP  Time-varying diastolic BP  Index age  Time-varying history of diabetes  Time-varying prescription of statins  Receipt of oral vitamin K  Receipt of 2 or more units of RBCs |
| 2013-2020 | 2008-2012 | Death, 45 days | Time-varying systolic BP  Time-varying diastolic BP  Index age  Time-varying history of dementia  Baseline history of intracranial bleeds  Time-varying history of diabetes  Time-varying history of chronic lung disease   Time-varying prescription of calcium channel blockers  Time-varying prescription of statins  Receipt of oral vitamin K  Receipt of 2 or more units of RBCs |
| 2013-2020 | 2013-2020 | Confirmed TEE, 45 days | Low income by census  Baseline history of acute MI |
| 2013-2020 | 2013-2020 | Death, 45 days | Index age  Time-varying history of diabetes  Time-varying history of heart failure  Time-varying prescription of alpha ARAs  Time-varying prescription of statins  Follow-up prescription of VKA or anticoagulants  Receipt of 2 or more units of RBCs |
